# World Hepatitis Day in 2022: Challenges of Viral Hepatitis Elimination in Elongated COVID-19 Pandemic

**DOI:** 10.3390/pathogens11091002

**Published:** 2022-09-01

**Authors:** Mahmoud Reza Pourkarim

**Affiliations:** 1 Laboratory for Clinical and Epidemiological Virology, Department of Microbiology, Immunology and Transplantation, Rega Institute, KU Leuven, 3000 Leuven, Belgium; mahmoudreza.pourkarim@kuleuven.be; 2 Health Policy Research Centre, Institute of Health, Shiraz University of Medical Sciences, Shiraz P.O. Box 71348-14336, Iran; 3 Blood Transfusion Research Centre, High Institute for Research and Education in Transfusion Medicine, Tehran 14665-1157, Iran

Recently, the World Hepatitis Day (WHD) of 2022 was observed to raise awareness of the global burden of viral hepatitis. Ambitious goals have been set by the WHO viral hepatitis elimination program in 2016. However, global issues of war, climate change, and the devastating SARS-CoV-2 pandemic challenge the success of the viral hepatitis elimination program.

A 90% global reduction in new infections and 60% reduction in viral hepatitis-related mortality by 2030 are the ultimate goals of this elimination program [1]. The major burden of viral hepatitis is caused by hepatitis B virus (HBV) and hepatitis C virus (HCV) [2].

Accordingly, the efficient vaccine against HBV infection and effective antiviral therapy for HCV are key interventions in the elimination program. Furthermore, the prevention of mother-to-child transmission by providing antiviral treatment to viremic pregnant mothers and the implementation universal vaccination of newborns are critical strategies to reduce HBV cases [3]. Additionally, screening of blood donors for both HBV and HCV plus safe injection practices are two important prophylactic interventions [4]. Finally, a careful follow-up of diagnosed cases in clinical practices (called linkage to care) is considered the final attempt to halt an infection cycle. Experts in the field of viral hepatitis and public health policymakers believe that these strategies are currently confronted with critical challenges caused by global changes [2].

Despite some regional improvements, the current annual death rate (1.34 million) and number of chronic viral hepatitis carriers (350 million) indicate that major steps still have to be taken. In addition, a recent report estimated 1.5 million new HBV infections and 820,000 corresponding deaths in 2019, three years after launching the elimination program [2,5]. Multiple reasons can be addressed that might hamper the success of the elimination program.

Technical limitations are one of the issues that undermine the goals of the elimination program. For instance, access to viral hepatitis screening tests is not optimal in several countries, and only 10% of chronic carriers have been diagnosed [5]. In African countries where more than one-quarter of viral hepatitis carriers are living, access to diagnostic services is still very difficult. Recent estimations show that almost two-thirds of the world’s HBsAg-positive children (below 5 years) inhabit Africa [6]. Together with the lack of access to screening tests for pregnant women and weak public knowledge about the risk factors of viral hepatitis infection, these limitations can have serious consequences for the viral hepatitis burden in this region [7].

Although several epidemiological studies for viral hepatitis have been performed [8] and different strategies for diagnosis [9], treatment [10], and curb of local outbreaks have been [11] developed, comprehensive epidemiological studies [5] are still lacking and post-prophylactic interventions are not well evaluated [2,12].

In addition to technical deficiencies, the limited funding is a major problem that confronts the elimination program. Compared to other infectious diseases, the viral hepatitis-specific funding is globally insufficient [13]. Furthermore, dedicated funding in health programs of individual countries has not been implemented on a large scale.

The emerging SARS-CoV-2 pandemic has greatly affected the direction of research efforts in the medical field. The pandemic is still ongoing and massively grips global and regional health-related funds. Billions of dollars that could support surveillance and healthcare services related to viral hepatitis are now being invested to suppress the COVID-19 pandemic [2,14]. A survey among 32 European and 12 non-European countries showed that the economic downturn of the COVID-19 pandemic has a negative impact on the rate of viral hepatitis testing and HBV vaccinations [15]. Since these are key tools in the elimination program, countries should invest in reaching pre-pandemic levels of prophylactic interventions.

In recent years, climate change has sharply intensified, and the world population is suffering from natural disasters such as floods, earthquakes, drought, and famine. Variations in climate parameters elevate the chance of transmission of water-borne pathogens such as noroviruses [16], rotaviruses [17], hepatitis A virus (HAV), and hepatitis E virus (HEV). Both hepatitis viruses also contribute to the global burden of viral hepatitis and should therefore be carefully monitored in light of the changing climate [18].

In addition to climate change, the world has been affected by the conflict between Ukraine and Russia, which jeopardizes the stability of the world economy and subsequently the global health budget. Furthermore, access to healthcare facilities for all war-affected areas is challenging [19,20], and the treatment of HBV and HCV has dramatically declined in these regions [15].

Needless to say, both climate change and the war in Ukraine have caused a wave of immigrants, forming a large reservoir of viral hepatitis carriers from regions with high prevalence to regions with low prevalence. Control of viral hepatitis in these moving populations needs immediate financial support and tailored health strategies authorized by destination countries [11,21,22].

Financial funding for the implementation of the viral-hepatitis-elimination program is not available in several countries [23]. Mostly, these are countries of low and middle income, although several studies have shown that even high-income countries are not on track with HCV treatment and HBV prophylaxis [2,23]. Recent evaluations show that only 11 out of 66 high-income countries are on track to eliminate viral hepatitis. Therefore, independent of the economic situation, all global efforts to reach the defined goals are still insufficient [24].

We can conclude that because of technical and financial issues, the viral hepatitis elimination program is being held back, and adhering to its goals by 2030 seems unlikely. Several important gaps, including public awareness and financial and health services deficiencies in most continents, still exist, and without solving them, tackling viral hepatitis at the global level is impossible. Based on the current status, the action plans should be revised and reconsidered. For instance, focusing on the most vulnerable populations (such as multi-transfused, people who use drugs, prisoners, and immigrants [12,25,26]) in the frame of micro-elimination [27], together with multiple key interventions, seems more feasible at this moment. Of note, a close collaboration between key stakeholders, and academic and non-academic organizations in each region can lead to realistic, community-based, and tailored strategies deployment. Indeed, promoting sustained attention on new communicative platforms supports better leverage of public awareness [28].
